# The Relationship between Antimicrobial Use and Antimicrobial Resistance in Europe

**DOI:** 10.3201/eid0803.010192

**Published:** 2002-03

**Authors:** Stef L.A.M. Bronzwaer, Otto Cars, Udo Buchholz, Sigvard Mölstad, Wim Goettsch, Irene K. Veldhuijzen, Jacob L. Kool, Marc J.W. Sprenger, John E. Degener, participants in the European Antimicrobial Resistance Surveillance System

**Affiliations:** * National Institute of Public Health and the Environment, Bilthoven, the Netherlands; † Swedish Institute for Infectious Disease Control, Solna, Sweden; ‡ The Nepi Foundation, Malmö, Sweden; § Groningen University Hospital, Groningen, the Netherlands

**Keywords:** antimicrobial resistance, penicillin nonsusceptible *S. pneumoniae*, antimicrobial use, ecologic study

## Abstract

In Europe, antimicrobial resistance has been monitored since 1998 by the European Antimicrobial Resistance Surveillance System (EARSS). We examined the relationship between penicillin nonsusceptibility of invasive isolates of *Streptococcus pneumoniae* (an indicator organism) and antibiotic sales. Information was collected on 1998-99 resistance data for invasive isolates of *S. pneumoniae* to penicillin, based on surveillance data from EARSS and on outpatient sales during 1997 for beta-lactam antibiotics and macrolides. Our results show that in Europe antimicrobial resistance is correlated with use of beta-lactam antibiotics and macrolides.

Antimicrobial resistance is a growing problem worldwide, requiring international approaches. The World Health Organization (WHO) and the European Commission have recognized the importance of studying the emergence and determinants of resistance and the need for strategies for its control (*1*–*3*). In European countries, antimicrobial resistance has been monitored in selected bacteria from humans since 1998 through the European Antimicrobial Resistance Surveillance System (EARSS). Funded by the European Commission, EARSS is an international network of national surveillance systems intended to collect comparable and reliable resistance data. The purpose of EARSS is to document variations in antimicrobial resistance over time and place and to provide the basis for and assess the effectiveness of prevention programs and policy decisions.

One of the indicator organisms in EARSS is *Streptococcus pneumoniae*. It was included for three reasons: it is of major clinical importance for pneumonia, bacterial meningitis, and otitis media; many countries have reported that its resistance to penicillin is increasing; and *S. pneumoniae* is representative of organisms that are transmitted in the community.

A major risk factor for the development of resistance is thought to be inappropriate use of antimicrobial drugs. Most studies that have investigated the relationship of antimicrobial use and antimicrobial resistance have been undertaken in hospital, multicenter, or country settings (*4*–*7*). For infections with penicillin-nonsusceptible *S. pneumoniae* (PNSP), studies have demonstrated that at the individual level, previous use of beta-lactam antibiotics such as penicillin is an important risk factor (*8*–*10*). Studies on carriage of PNSP in children have shown that sulfamethoxazole-trimethoprim (co-trimoxazole) and macrolides such as erythromycin have also been associated with selection of PNSP (*11*,*12*). Translated to the population level, sales of beta-lactam antibiotics, co-trimoxazole, or macrolides in a given geographic region may be proportional to microbial resistance to penicillin. If on the European level a relationship between antimicrobial resistance and antimicrobial use could be found (as in the case of *S. pneumoniae* and resistance to penicillin), efforts to control antimicrobial use and misuse could be stimulated and monitored in Europe.

We used an ecologic study design to examine the correlation between use of relevant antibiotics in the outpatient setting and the proportion of PNSP among invasive isolates of *S. pneumoniae* in 11 European countries.

## Methods

### Antimicrobial Resistance Data

The estimated average coverage of the populations of countries participating in EARSS is 52% (range 10% to 90%) (*13*). Laboratories that participate in EARSS screen invasive *S. pneumoniae* isolates for oxacillin resistance (*14*). When an isolate is found to be nonsusceptible, the EARSS protocol requests confirmation as intermediate- or high-level resistance to penicillin by determination of MICs. Laboratories perform microbiologic testing and interpret results according to their own standards. National guidelines in Europe differ; isolates of *S. pneumoniae* are considered nonsusceptible to penicillin if the MIC is >0.06 (*15*–*18*) or >0.12 (*19*,*20*) mg/L. For this report, we use nonsusceptibility and intermediate resistance as synonyms; PNSP isolates are either intermediate or fully resistant to penicillin. Only the first invasive isolate per patient per quarter is reported.

To assess the comparability of susceptibility test results, a quality assurance exercise was performed in September 2000 among 482 laboratories from 23 countries participating in EARSS. The concordance (agreement of reported results with intended results) for the detection of penicillin resistance in the three *S. pneumoniae* control strains was 91% (*21*). Laboratories sent standardized data to the national EARSS data manager, who checks data contents and ensures conformity with the EARSS data format. In collaboration with WHO, an export module from the laboratory-based software WHONET was developed for EARSS (*22*). Every quarter, data are forwarded to the central database at the National Institute of Public Health and the Environment (RIVM), Bilthoven, Netherlands, where the project is coordinated.

### Antimicrobial Use Data

National outpatient sales data for antibiotics from 1997 were purchased from IMS Health Global Services, London, United Kingdom, for 13 of the 15 member states of the European Union. Corresponding data were obtained from the Danish Medicines Agency for Denmark and from the National Corporation of Swedish Pharmacies for Sweden (*23*). The IMS data were examined and adjusted according to the Anatomic Therapeutic Classification (ATC) system used by WHO (*24*). The amount in kilograms for an antimicrobial agent was converted to a number of defined daily doses (DDD). The DDD, which is based on the average daily dose used for the main indication of the drug, is appropriate for comparisons of drug use over time and in different geographic areas. For beta-lactam antibiotics, we combined ATC groups J01C (extended- and narrow-spectrum penicillins) and J01D (cephalosporins); macrolides were classified under code J01F. No data were available for the combination of trimethoprim and sulphonamide.

### Nonadherence

We considered nonadherence of patients to the physician’s prescription in individual countries as a possible confounder of antimicrobial resistance. Branthwaite et al. reported nonadherence levels from a population-based survey in seven countries (*25*). Data from four of the seven countries (Spain, Belgium, the United Kingdom, and Italy) were also captured in EARSS.

### Statistical Analysis

We calculated the proportion of PNSP among all invasive *S. pneumoniae* isolates from each country reported during 1998-99. Because probabilities allow only values between 0 and 1, we modeled the natural logarithm of the odds of PNSP resistance (logodds).

Least-square linear regression analysis was used to assess correlation between antimicrobial use (of beta-lactam antibiotics and macrolides, expressed in DDD per 1,000 population per day) and the logodds of resistance. We correlated nonadherence levels with the logodds of resistance in the same way. We calculated the Spearman coefficient of determination (r-square) and its corresponding p value. For the calculation of the regression lines, we weighted the data points by the inverse of the variance of each data point. We used SAS software (SAS Institute Inc., Release 6.03., Cary, NC).

## Results

### Antimicrobial Resistance

**Figure 1:**
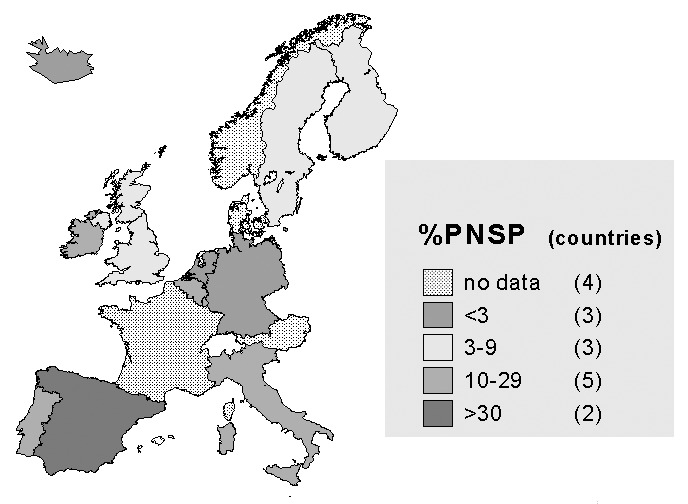
Proportions of invasive isolates of *Streptococcus pneumoniae* resistant to penicillin (PNSP) among 12 European countries, 1998-99.

During 1998-99, 337 laboratories from 11 European Union member states (Belgium, Finland, Germany, Ireland, Italy, Luxembourg, Netherlands, Portugal, Spain, Sweden, and United Kingdom) and one nonmember state (Iceland) reported 4,872 invasive *S. pneumoniae* isolates to EARSS. The proportion of PNSP among isolates of invasive *S. pneumoniae* ranged from 1% to 34% (Table) (Figure 1). Southern European countries reported higher rates than northern European countries.

**Table:** Number of submitting laboratories, number of isolates of *Streptococcus pneumoniae*, number (#) and percent (%R) of penicillin nonsusceptible *S. pneumoniae* isolates, logodds of resistance (ln(%R/[1-%R]), and outpatient sales of beta-lactam antibiotics and macrolides

Country	No. of labora-tories	No. of *S. pneumoniae* isolates	Penicillin nonsusceptible *S. pneumoniae*			Outpatient sales of antibiotics in DDD^a^/1,000 inhabitants/day	
			No.	%R (95% CI)	ln (%R/[1-%R])	Beta-lactam antibiotics	Macrolides
Austria	-	-	-	-	-	6	3.7
Belgium	96	940	131	14 (12-16)	-1.82	14	4.1
Denmark	-	-	-	-	-	7	2
Finland	11	211	8	4 (2-8)	-3.18	8[REMOVED ADVANCE FIELD]	[REMOVED ADVANCE FIELD]1.9
[REMOVED ADVANCE FIELD]**France**	[REMOVED ADVANCE FIELD]-	[REMOVED ADVANCE FIELD]-	[REMOVED ADVANCE FIELD]-	[REMOVED ADVANCE FIELD]-	[REMOVED ADVANCE FIELD]-	24[REMOVED ADVANCE FIELD]	[REMOVED ADVANCE FIELD]6.0
Germany	15	222	4	2 (1-5)	-3.89	5	2.5
Iceland	2	54	1	2 (0-11)	-3.89	Not available	Not available
Ireland	12	157	30	19 (13-26)	-1.45	11	2.5
Italy	46	194	26	13 (9-19)	-1.87	15	5.1
Luxembourg	1	11	2	18 (3-52)	-1.52	14	4.7
Netherlands	20	760	8	1 (0-2)	-4.6	4	1.2
Portugal	12	134	25	19 (13-27)	-1.45	16	3.7
Spain	76	1,240	418	34 (31-36)	-0.66	21	5.9
Sweden	24	706	21	3 (2-5)	-3.48	8	1
United Kingdom	22	243	21	9 (6-13)	-2.31	9	3.2

^a^DDD = defined daily doses; CI = confidence interval.

### Antimicrobial Use

Data on outpatient sales of beta-lactam antibiotics and macrolides were available for 1997 from all 15 European Union member countries. Antimicrobial use varied widely between countries. Sales to outpatients ranged from 3.8 to 23.6 DDD per 1,000 inhabitants per day for beta-lactam antibiotics and from 0.97 to 5.98 DDD for macrolides. The three countries with the highest reported use were France, Spain, and Portugal for beta-lactam antibiotics and France, Spain, and Italy for macrolides; the three countries with the lowest use were the Netherlands, Germany, and Austria for beta-lactam antibiotics and Sweden, the Netherlands, and Finland for macrolides.

### Correlation

**Figure 2:**
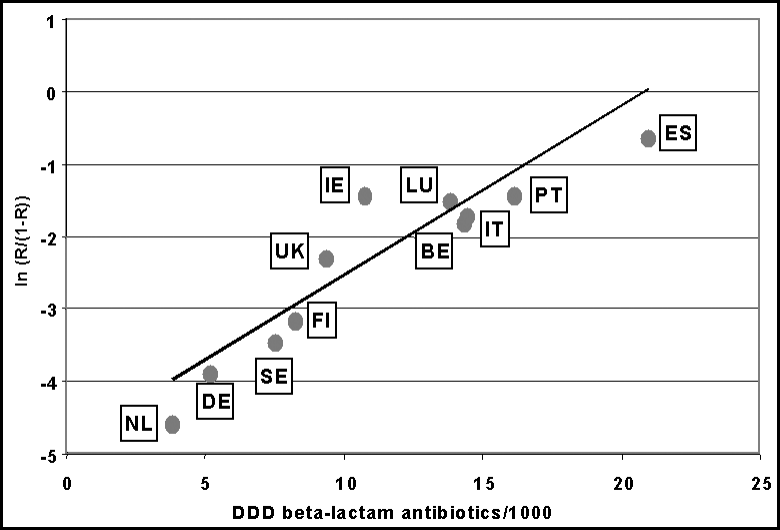
The logodds of resistance to penicillin among invasive isolates of *Streptoccus pneumoniae* (PNSP; ln(R/[1-R])) is regressed against outpatient sales of beta-lactam antibiotics in 11 European countries; antimicrobial resistance data are from 1998 to 1999 and antibiotic sales data are from 1997. DDD = defined daily dose; BE = Belgium; DE = Germany; FI = Finland; IE = Ireland; IT = Italy; LU = Luxembourg; NL = the Netherlands; PT = Portugal; ES = Spain; Se = Sweden; UK = United Kingdom.

For 11 countries, information was available for both antimicrobial resistance and antimicrobial use. Linear regression of the correlation of use of beta-lactam antibiotics and the logodds of resistance showed an r-square of 0.80 (p=0.0002) (Figure 2). The equation for the regression is

logodds of resistance =(-3.94)+(0.16xDDD).

**Figure 3:**
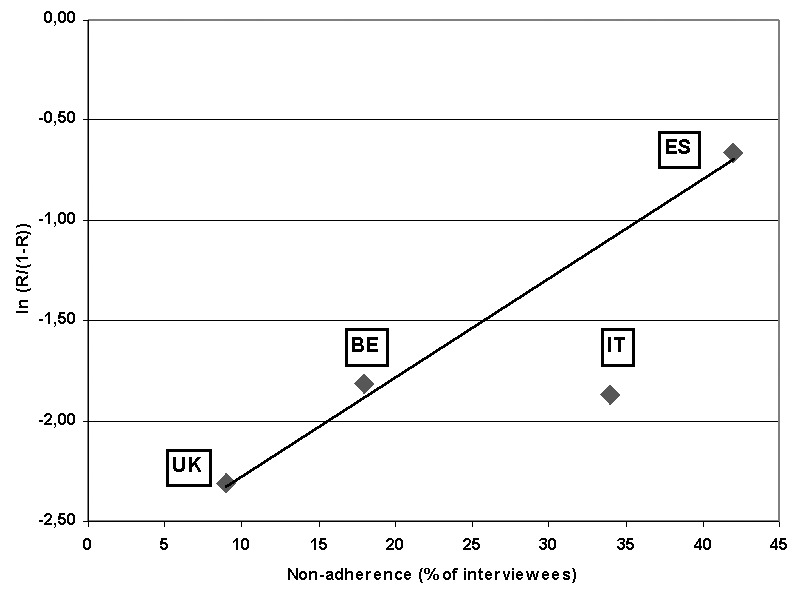
The logodds of resistance of invasive isolates of *Streptococcus pneumoniae* to penicillin (PNSP; ln(R/(1-R))) is regressed against nonadherence rates to antibiotic therapy in four European countries. Nonadherence rates are from 1993; PNSP data are from 1998-99. UK = United Kingdom; BE = Belgium; IT = Italy; ES = Spain.

For the use of macrolides, we calculated an r-square of 0.46. Figure 3 shows the graph for nonadherence to antibiotics and the logodds of resistance. The r-square is 0.8 (p=0.2).

## Discussion

We present for the first time Europe-wide, country-specific, representative data on antimicrobial resistance collected by EARSS. Using an ecologic study design, we demonstrate through the correlation with data on antimicrobial use one aspect of the usefulness of surveillance for antimicrobial resistance. The results from 11 European countries show a linear relationship between use of beta-lactam antibiotics and macrolides and the proportion of PNSP among all invasive *S. pneumoniae* isolates.

EARSS data show that resistance for PNSP follows a north-south gradient. Southern European countries have higher proportions of PNSP than countries in northern Europe. A possible reason for this observation could be the difference in antimicrobial use, which also tends to be higher in southern European countries. If use of relevant antibiotics (beta-lactam antibiotics and macrolides) and the logodds of resistance are modeled through linear regression, a strong linear and statistically significant relationship is demonstrated.

Our findings agree with those of Austin et al., who modeled the relationship between antimicrobial use and endemic resistance, based on population genetic methods and epidemiologic observations (*26*). The correlation in Figure 2 is consistent with the model developed by Austin et al. on theoretical grounds.

We correlate antimicrobial sales data for 1997 with antimicrobial resistance data for 1998 and 1999. Others have observed that after a lag time of 1 or more years, changes in antimicrobial use may be followed by changes in antimicrobial resistance (*27*,*28*). Therefore, we believe that it is reasonable to correlate antimicrobial sales data in 1997 with antimicrobial resistance data from 1998-99.

We address several limitations in our study. First, because it is an ecologic study, we can make no inferences on the individual level. Second, resistance rates in some countries (Table) are calculated from a relatively limited number of isolates. However, based on communications with EARSS country representatives, our data are consistent with antimicrobial resistance levels derived from other sources (*29*). Third, an explanation for the differences in antimicrobial resistance could be sampling bias: clinicians in southern European countries may request blood cultures more frequently than their northern European colleagues, who may sample only in case of empirical treatment failure. Fourth, we have not addressed other, potentially important contributing factors for the development of antimicrobial resistance of organisms that are transmitted in the community, particularly nonadherence and over-the-counter sales of antimicrobial agents. Both these factors are difficult to measure. However, in 1993 nonadherence to prescribed antimicrobial agents was assessed in a survey in six European countries (*25*). Although the number of data points is limited, Figure 2 suggests a direct relationship between nonadherence rates and logodds of resistance. Thus, if nonadherence is also related to sales of antimicrobial agents, it could potentially confound the relationship between use and resistance. Data on the degree of over-the-counter use among European countries are not widely available; we know of one Spanish and one Greek study reporting an estimate of over-the-counter use (*30*,*31*). The influence of these and other parameters on the level of resistance should be quantified and understood. Finally, because children are the main reservoir of carriage of *S. pneumoniae*, an age-stratified analysis would be desirable, i.e., a correlation of resistance with antimicrobial use among children. However, this analysis would require more detailed use data, for example, of liquid formulations of antibiotics.

At least two studies in northern Europe have demonstrated that PNSP rates can be halted or even reversed when physicians avoid the inappropriate prescription of antimicrobial agents (*32*,*33*). Our study is timely because it shows that even at the European level a correlation can be observed between antimicrobial resistance (of *S. pneumoniae* to penicillin) and antimicrobial use. In several European countries, national action plans for the appropriate use of antimicrobial agents are being planned or implemented; their effectiveness should be monitored through prospective and continuous surveillance of antimicrobial resistance and antimicrobial sales data (*34*–*38*).
